# Elucidating the Effect of Endophytic Entomopathogenic Fungi on Bread Wheat Growth through Signaling of Immune Response-Related Hormones

**DOI:** 10.1128/aem.00882-22

**Published:** 2022-08-29

**Authors:** Adrián González-Guzmán, María-Dolores Rey, Emilie Froussart, Enrique Quesada-Moraga

**Affiliations:** a Department of Agronomy, ETSIAM, University of Cordoba, Córdoba, Spain; b Agroforestry and Plant Biochemistry, Proteomics and Systems Biology, Department of Biochemistry and Molecular Biology, University of Cordoba, UCO-CeiA3, Cordoba, Spain; c Department of Plant Biotechnology and Bioinformatics, Ghent University, Ghent, Belgium; d Center of Plant Systems Biology, VIB, Ghent, Belgium; Royal Botanic Gardens

**Keywords:** *Beauveria bassiana*, *Metarhizium brunneum*, induced systemic resistance, plant hormone, RT-qPCR, plant-microbe interaction

## Abstract

Entomopathogenic fungi (EF) provide a potent biocontrol tool; also, their endophytic behavior has broadened their contribution to integrated pest management (IPM) and crop production. In this work, Beauveria bassiana and Metarhizium brunneum were applied to bread wheat (Triticum aestivum) seedlings to elucidate how fungal colonization influences plant growth and the relative expression of 24 genes involved in hormonal syntheses and plant immune mechanisms. A preliminary assay was used to determine the time needed for fungal colonization and assess its effect on wheat growth. Then, plant material collected at various times after inoculation (*viz.*, 2, 8, 20, and 36 h and 9 and 15 days) was used to investigate gene expression by quantitative reverse transcription PCR (RT-qPCR). During the colonization time, B. bassiana and M. brunneum caused strong downregulation of most genes associated with plant immunity and the synthesis of hormones like auxin, cytokinin, and gibberellin. This effect was concomitant with a slowdown of endophytic-colonization-related plant growth until 19 days postinoculation (dpi). However, the wheat started to recover at 15 dpi, simultaneously with upregulation of auxin- and gibberellin-related genes. The results suggest that the EF trigger induced systemic resistance rather than acquired systemic resistance during early plant-microbe cross talk in wheat. Also, they confirm that the hormone and immune responses of wheat triggered by EF inoculation influenced plant growth, which can be useful with a view to optimizing management of these microorganisms for sustainable agriculture.

**IMPORTANCE** Microbial control of insect and mite pests is a key tool to develop integrated pest management (IPM) and sustainable agriculture. Entomopathogenic fungi (EF) may have associations with the plants, playing additional ecological roles in the rhizosphere, in the phylloplane, and as plant endophytes. Beauveria bassiana 04/01TIP and Metarhizium brunneum 01/58Su are two strains that showed very good results either in pest control or plant growth promotion and would be good candidates to develop mycoinsecticides as an alternative to pesticides. However, deep knowledge about their interaction with the plant would let farmers optimize their use and understand the plant response, enhancing and promoting their broader contribution to IPM and crop production.

## INTRODUCTION

The global population is expected to increase to 9.9 billion by 2050 and hence to raise the need for increased crop production (1, 2). This will also increase the use of agricultural chemicals with an environmental impact, such as fertilizers and pesticides (3, 4). As a result, developing new, effective strategies for crop production and protection will become a major challenge for agricultural sustainability (5). The new strategies should include the use of soil microorganisms that colonize plant roots, which has already proved effective for ensuring sustainability in agricultural ecosystems (6–9) by enhancing plant nutrient uptake, stimulating plant growth, or alleviating biotic and abiotic stresses (5, 6, 10–13).

Entomopathogenic fungi (EF) are a group of microorganisms used as biological control agents to replace pesticides in a number of agroecosystems (5, 14–18). Moreover, their endophytic behavior has broadened their contribution to crop production through improved plant responses to other biotic (e.g., plant disease) and abiotic stresses (19–22). Senthilraja et al. (23) found EF inoculation to protect against future pest attacks through “plant priming” resulting from the prior triggering of induced systemic resistance (ISR). They found the presence of EF to boost production of pathogenesis-related proteins, increase enzyme activity induced by biotic and abiotic stresses, and trigger immune-related genes. Only a few studies, however, have focused on ISR triggering by EF (23–25). Mycosynergism with an axenic consortium of B. bassiana and Trichoderma asperellum against Ostrinia furnacalis was found to boost herbivory-induced maize defense by triggering antioxidant and phytohormone signaling in the framework of comprehensive transcriptome and untargeted metabolome profiling in fungally inoculated maize leaves. Indeed, the output of a weighted gene coexpression network analysis using 13,156 differentially expressed genes revealed six significant modules containing 13 candidate genes that have not been previously reported to be highly correlated with the jasmonic acid-ethylene (JA/ET) signaling pathway and antioxidants (26). The consortium considerably raised jasmonic acid, salicylic acid (SA), and ethylene levels (26), as seen in previous studies (27). Hence, crop colonization by EF may boost herbivory-induced defenses and restrict pest survival and growth as a result.

Some recent studies have shown the EF Beauveria bassiana and Metarhizium brunneum to considerably reduce root length, plant height, and dry matter contents at the initial growth stage of wheat (Triticum aestivum) (30 on Zadok’s growth scale) (28, 29); the effects, however, vanished at stem elongation and heading on Zadok’s growth scale (30). Therefore, it is necessary to research the relationship of molecular hormone metabolism in the plant immune system with either physiological changes or EF colonization, both of which remain unexplored but are key points to enhance the use of these microorganisms.

In this work, the starting hypothesis was that triggering of the plant immune system was directly responsible for the impact of fungal colonization at an early plant growth stage, when plant-fungus cross talk was crucial for rapid colonization and subsequent growth promotion. To address this hypothesis, the time needed for EF colonization and its effect on bread wheat growth and yield were determined. We used 3-day-old wheat seedlings inoculated with either B. bassiana or M. brunneum to elucidate hormonal cross talk and signaling during fungal colonization of the crop at the initial growth stage. For this purpose, reverse transcription-quantitative PCR (RT-qPCR) was used to assess the relative expression levels of genes involved in synthetic, catabolic, and hormone signaling pathways to account for the observed effects on bread wheat.

## RESULTS

### Seedling colonization time and rate.

Both B. bassiana and M. brunneum colonized wheat seedlings by up to 66% (4 out of 6 plants). In the first experiment, colonization by M. brunneum increased with time (0, 33, and 66% at 12, 30, and 48 h postinoculation [hpi], respectively), whereas colonization by B. bassiana peaked at 30 hpi (Table 1). In the second experiment, the levels of B. bassiana and M. brunneum colonization at 36 hpi amounted to 50% and 66%, respectively.

**TABLE 1:** Percentages of fungal reisolation of B. bassiana and M. brunneum at the initial stage of seedling growth in each experiment

Treatment	% of plants colonized at indicated time (hpi) in^*a*^:				
	First expt			Second expt	
	12	30	48	12	36
B. bassiana	0	66	50	17	50
M. brunneum	0	33	66	0	66

a The control showed no fungal outgrowth (data not shown). hpi, hours postinfection.

### Effects of EF inoculation on plant height, dry matter, and root architecture.

**FIG 1:**
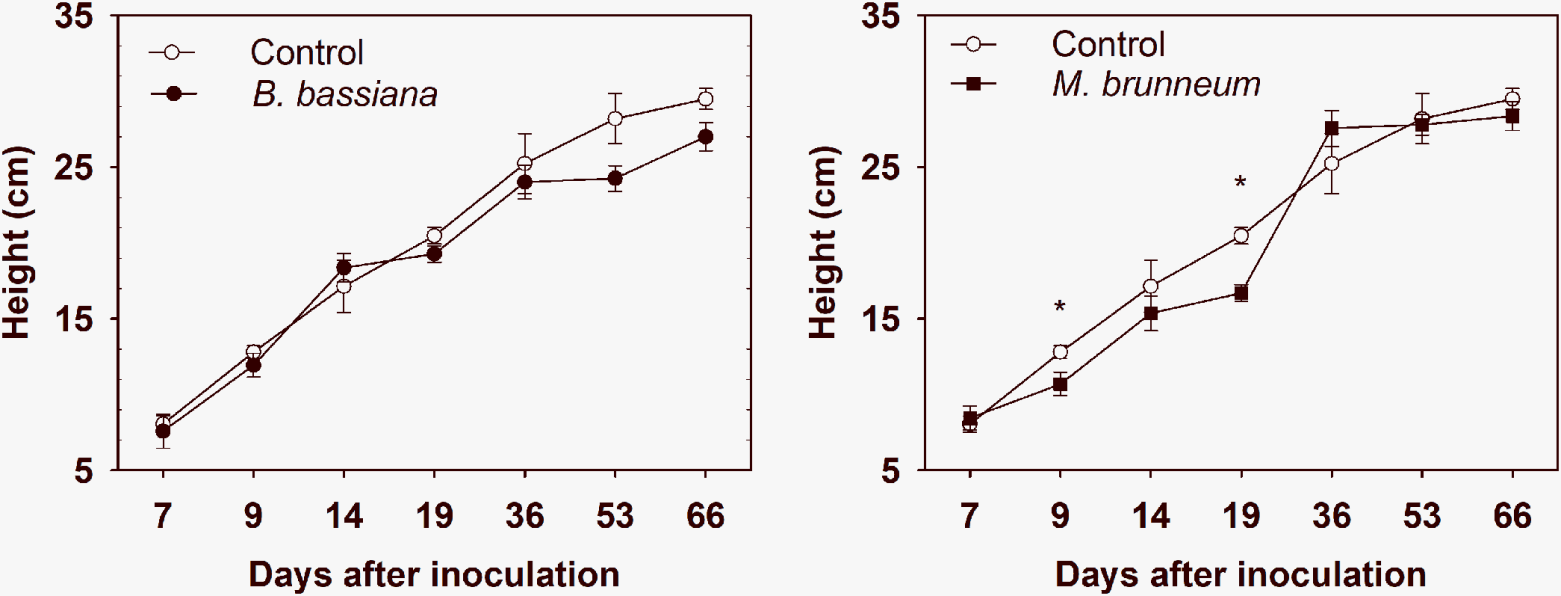
Variation of plant height with time. Error bars show standard errors. *, *P < *0.05 for significant differences between means.

Wheat plants inoculated with B. bassiana differed little in height from control plants (Fig. 1A). However, height in M. brunneum-treated plants exhibited significant decreases at 9 and 19 dpi but no difference at 36 dpi or thereafter (Fig. 1B).

**FIG 2:**
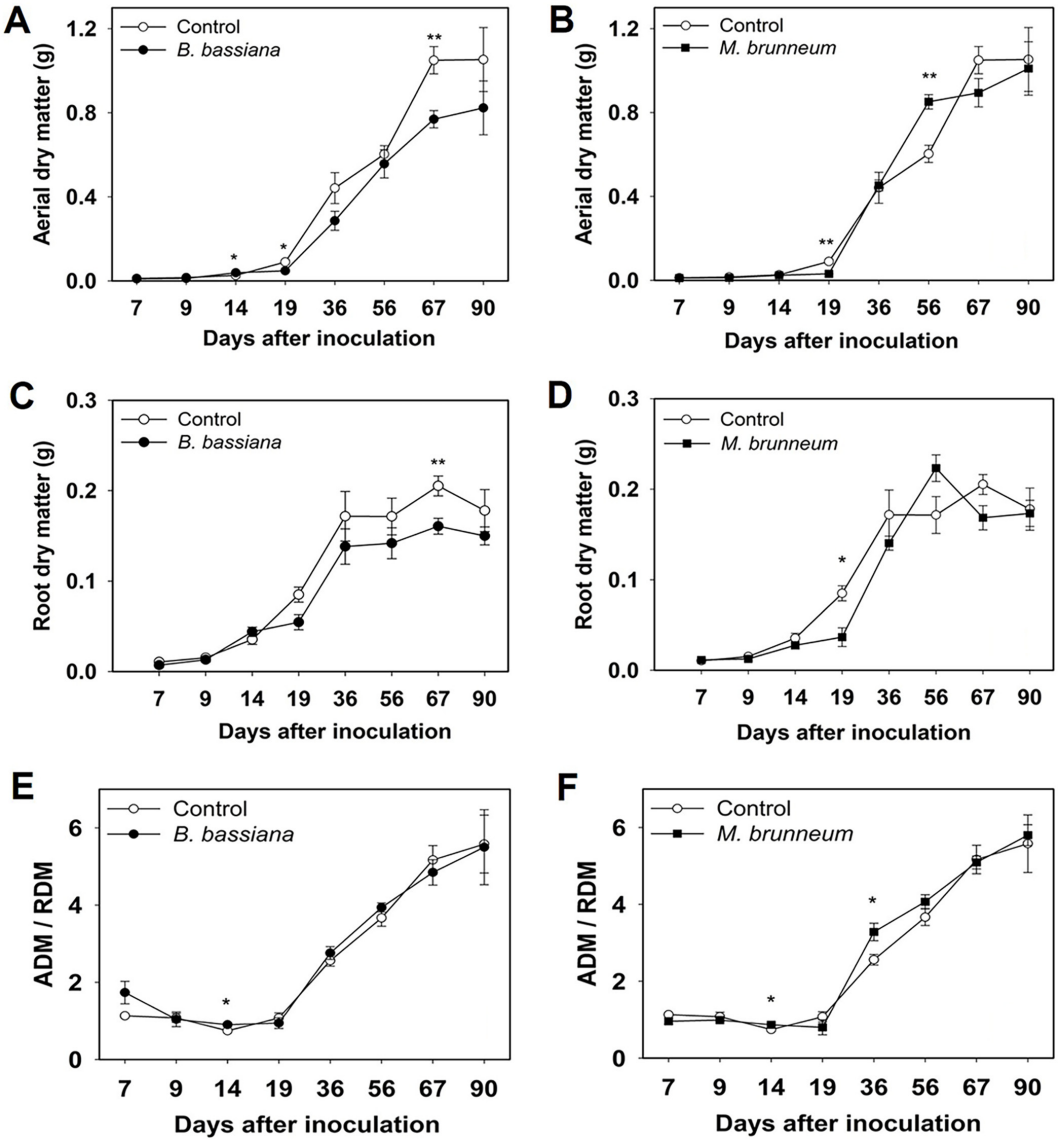
Variation of aerial and root biomass in plants treated with B. bassiana (A and C) and M. brunneum (B and D) and control plants with time. Variations of the aerial-to-root dry matter ratio (ADM/RDM) in the fungal and control treatments with time. (E and F) Error bars show standard errors. *, *P < *0.05.

Aerial dry matter (ADM) and root dry matter (RDM) showed punctual differences between fungal treatments. For example, the results showed significant increases in ADM 14 dpi with B. bassiana and 56 dpi with M. brunneum (Fig. 2A and B). Both EF additionally decreased ADM significantly at 19 dpi, and B. bassiana also decreased ADM at 67 dpi (Fig. 2A and B). Root dry matter (RDM) followed an identical trend, with significant decreases at 67 and 19 dpi in the plants treated with B. bassiana and M. brunneum, respectively (Fig. 2C and D). The ADM/RDM ratio was significantly increased by both fungi at 14 dpi (Fig. 2E and F) and also by M. brunneum at 36 dpi (Fig. 2E). No significant differences in grain yield were observed with either EF, however (Fig. S1 in the supplemental material).

**FIG 3:**
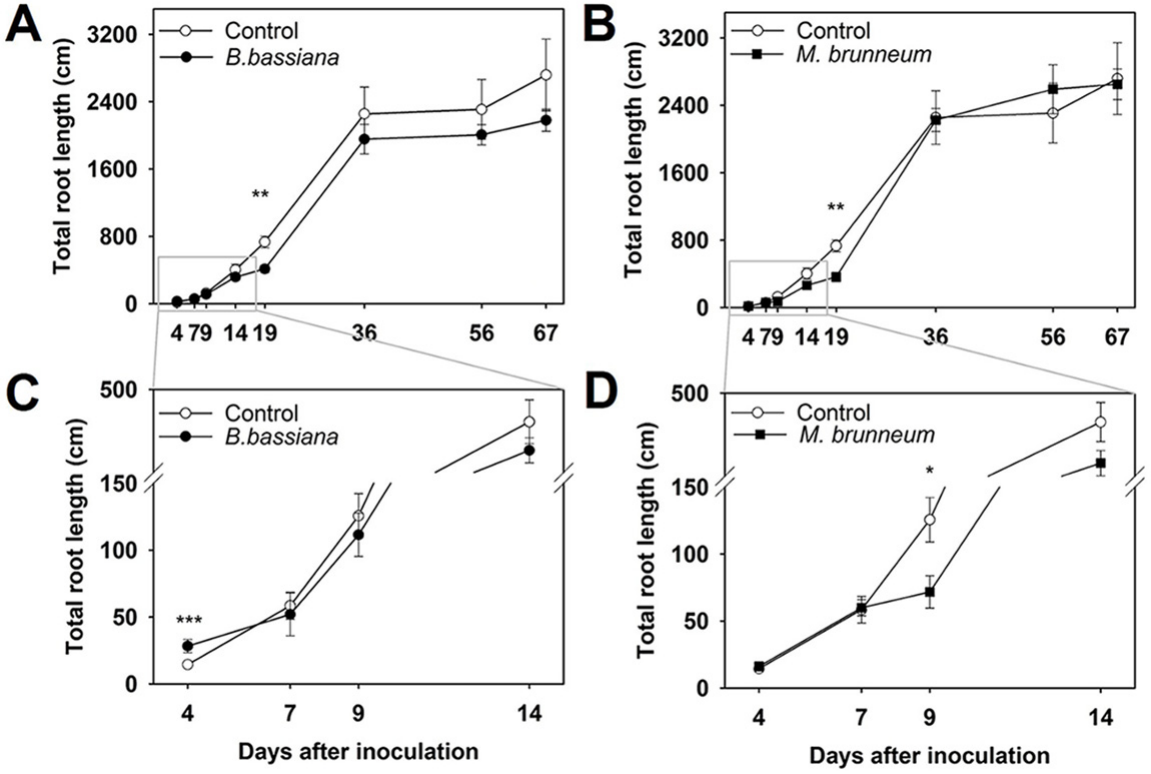
Variations of total root length (TRL) with time in the B. bassiana (A and C) and M. brunneum treatments (B and D) relative to the control treatment. Error bars show standard errors. *, *P < *0.05; **, *P < *0.01; ***, *P < *0.001.

Changes in root architecture were assessed in terms of total root length (TRL), root surface area (RSA), specific root length (SRL [RL/RDM]), and specific root area (SRA)—which followed the same trend as SRL (data not shown). TRL similarly evolved to ADM and RDM; thus, it was decreased by both fungal treatments relative to that in the control treatment (Fig. 3C and D), especially at 9 (M. brunneum) and 19 dpi (Fig. 3A and B). The differences, however, vanished later (Fig. 3A and B). An anecdotal significant increase with B. bassiana was also observed at 4 dpi (Fig. 3C).

The higher relative decrease of TRL with respect to RDM with both fungi resulted from the formation of a lesser amount of fine roots and, thus, a decrease in RSA (Fig. S2A and B). However, measurements of relative root lengths in terms of the amount of biomass invested by M. brunneum*-*treated plants revealed an increased efficiency from 14 dpi (Fig. S3).

### Expression patterns of marker genes involved in hormone signaling and immunity in wheat upon inoculation with EF. (i) RGE in the presence of B. bassiana.

**FIG 4:**
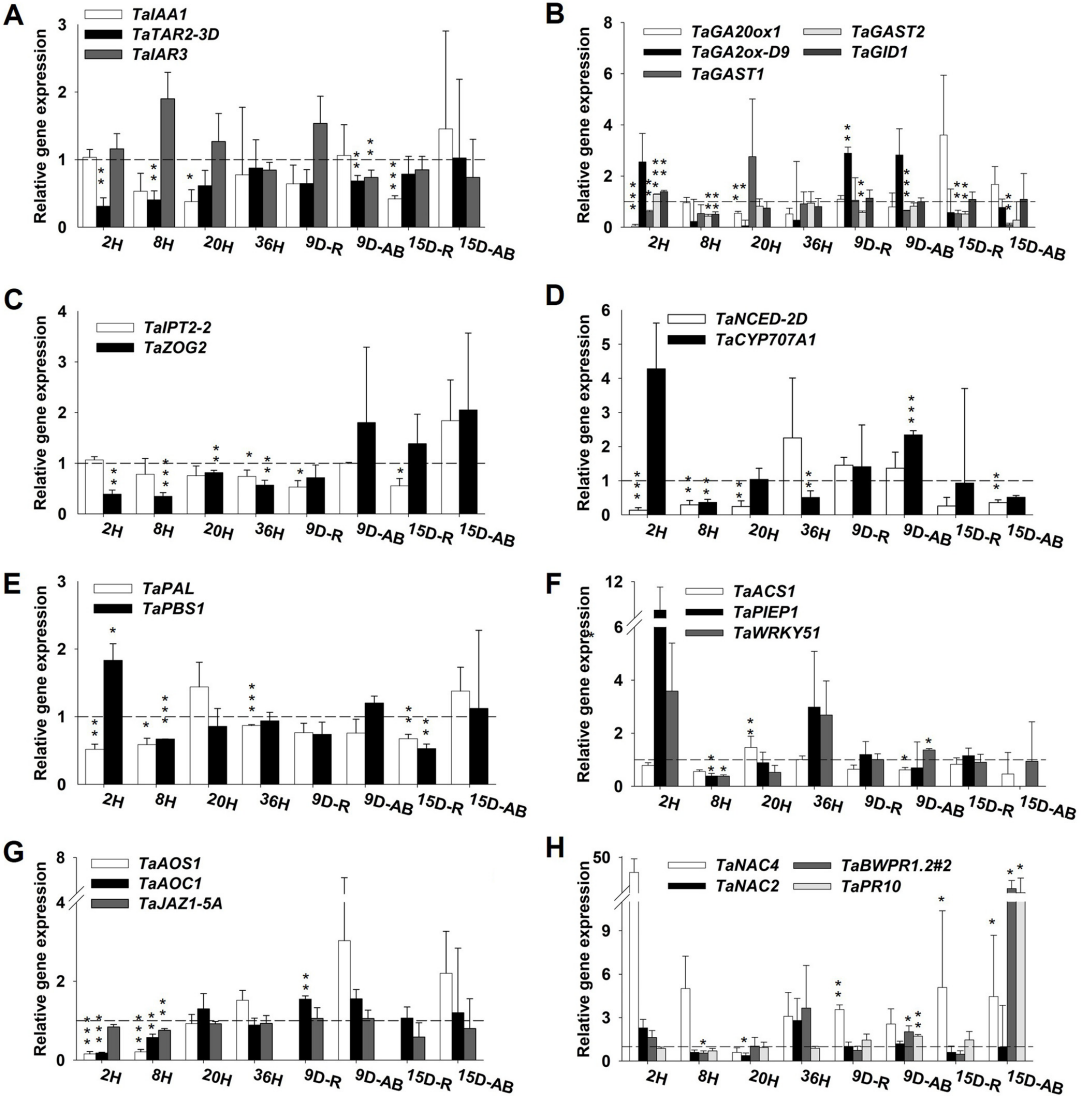
Relative gene expression profiling (normalization against control seedlings) of genes (see Table 2) by relationship with the hormones auxin (A), gibberellin (B), cytokinin (C), abscisic acid (D), salicylic acid (E), ethylene (F), jasmonate (G), and transcription factors and PR proteins involved in regulation of the immune system (H) in B. bassiana-treated seedlings. Timeline abbreviations: H, hours postinoculation; D, days postinoculation; R, roots; AB, aerial biomass. Error bars show standard errors. *, *P < *0.05; **, *P < *0.01; ***, *P < *0.001.

Figure 4 shows the relative gene expression (RGE) levels for 24 genes associated with phytohormones, transcriptional factors, and pathogenesis-related proteins (PR) in B. bassiana-treated and control seedlings. The RGE results (up- or downregulation relative to the expression in control plants) correspond to fungus penetration time (2 to 36 hpi) and also to 9 and 15 dpi for either root (R) or aerial biomass (AB).

Synthesis of auxin via the tryptophan pathway (*TaTAR2-3D*) in B. bassiana-treated seedlings was repressed throughout, and so was that of auxin-response protein (*TaIAA-1*); on the other hand, synthesis of *TaIAR3* was slightly upregulated at the beginning of fungal penetration and at 9 dpi in roots (Fig. 4A). Synthesis of gibberellins (GAs) was suppressed (*TaGA20ox1*) until 15 dpi, while degradation of GAs (*TaGA2ox-D9*) was higher at 2 hpi and then at 9 dpi (Fig. 4B). *TaGAST1*, *TaGAST2*, and *TaGID1* were generally downregulated and exhibited no substantial change. Biosynthesis of cytokinin (CK) hormone (*TaIPT2-2*) was repressed—significantly in roots—until 15 dpi in AB. The RGE of *TaZOG2* increased throughout; the gene was significantly downregulated during the colonization time but upregulated in AB from 9 dpi (Fig. 4C). Synthesis of abscisic acid (ABA) hormone (*TaNCED-2D*) was generally repressed (Fig. 4D). *TaNCED-2D* was significantly downregulated at the beginning of colonization but upregulated at 36 hpi, when it started to decrease again, following a Gaussian curve. *TaCYP707A1* was upregulated at 2 hpi and 9 dpi (Fig. 4D). The salicylic acid synthesis pathway (*TaPAL*) and RGE in *TaPBS1* were generally repressed, which highlighted the significant overexpression of *TaPBS1* at 2 hpi. Ethylene synthesis through *TaACS1* was upregulated at 20 hpi only. *TaPIEP1* and *TaWRKY51* were strongly upregulated at 2 and 36 hpi, whereas *TaWRKY51* was slightly but significantly upregulated at 9 dpi in AB (Fig. 4F). RGE in *TaAOS1* (not detected in roots) and *TaAOC1* (involved in jasmonate synthesis) also showed Gaussian distribution, both being significantly repressed until 20 hpi. On the other hand, RGE in *TaJAZ1-5A* remained unchanged (Fig. 4G). Transcriptional factors showed the greatest differences in RGE, *TaNAC4* being strongly upregulated at all times except at 20 hpi (Fig. 4H). In contrast, *TaNAC2* was downregulated at all times except at 2 and 36 hpi (Fig. 4H). Finally, the genes related to PR proteins (*TaBWPR1.2#2* and *TaPR10*) were upregulated after fungal penetration, but mainly in aerial biomass (Fig. 4H).

### (ii) RGE in the presence of M. brunneum.

**FIG 5:**
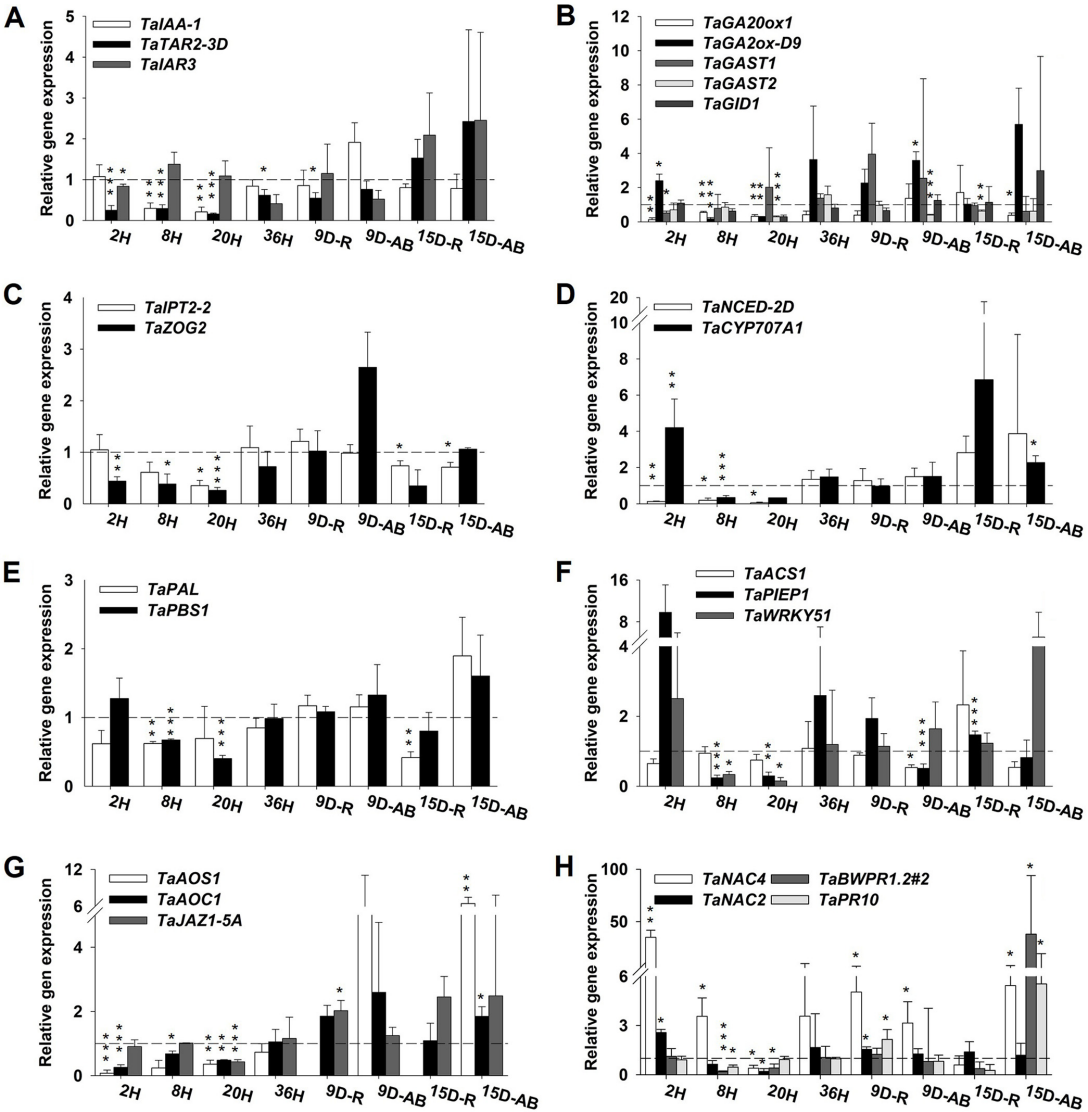
Relative gene expression profiling (normalization against control seedlings) of genes (see Table 2) by relationship with the hormones auxin (A), gibberellin (*TaGID1* and *TaGAST2* excepted) (B), cytokinin (C), abscisic acid (D), salicylic acid (E), ethylene (F), jasmonate (G), and transcription factors and PR proteins involved in regulation of the immune system (H) in M. brunneum-treated seedlings. Timeline abbreviations: H, hours postinoculation; D, days postinoculation; R, roots; AB, aerial biomass. Error bars show standard errors. *, *P < *0.05; **, *P < *0.01; ***, *P < *0.001.

Auxin synthesis in M. brunneum-treated seedlings was repressed (*TaTAR2-3D* was downregulated); however, RGE was markedly increased at 15 dpi for both *TaTAR2-3D* and *TaIAR3* (Fig. 5A). Consequently, *TaIAA-1* remained downregulated, albeit with no substantial changes (Fig. 5A). The synthesis of GA (*TaGA20ox1*) was repressed at fungal colonization time but slightly boosted in aerial biomass and roots at 9 and 15 dpi, respectively (Fig. 5B). The hormone exhibited high catabolic activity (upregulation of *TaGA2ox-D9*) except at 8 and 20 hpi (Fig. 5B). *TaGAST1*, *TaGAST2*, and *TaGID1* exhibited no appreciable changes in M. brunneum-treated seedlings or plants, the first gene being strongly upregulated at 9 dpi, with no differences between biological replicates due to the high standard errors (Fig. 5B). *TaIPT2-2* and *TaZOG2*, involved in cytokinin synthesis and metabolism, exhibited no significant differences in expression other than substantial downregulation at 8 (*TaZOG2* only) and 20 hpi (Fig. 5C). ABA biosynthesis (*TaNCED-2D*) increased gradually over time. *TaCYP707A1* was upregulated at 2 hpi and 15 dpi but significantly downregulated at 8 and 20 hpi (Fig. 5D). The expression of the genes associated with salicylic acid metabolism (*TaPAL* and *TaPBS1*) was essentially identical between treatments. Interestingly, there was considerable downregulation of *TaPAL* at 8 hpi and 15 dpi in roots, and also of *TaPBS1* at 8 and 20 hpi (Fig. 5E). As regards ethylene synthesis, *TaACS1* failed to respond to the presence of M. brunneum; in contrast, *TaPIEP1* and *TaWRKY51* were strongly upregulated at 2 hpi but significantly downregulated at 8 and 20 hpi until the end of fungal colonization at 36 hpi, when the upregulation of *TaPIEP1* was especially marked. Subsequently, *TaPIEP1* exhibited increased RGE in roots (R), as did *TaWRKY51* in aerial biomass (AB) (Fig. 5F). The expression of genes involved in jasmonate synthesis (*TaAOS1* and *TaAOC1*) increased throughout (Fig. 5G); both genes were downregulated until 36 hpi but upregulated thereafter. However, M. brunneum colonization resulted in upregulation of *TaJAZ1-5A* at 9 and 15 dpi, and also in anecdotal, significant downregulation at 20 hpi. The transcriptional factor *TaNAC4* was always upregulated except at 20 hpi and 15 dpi (roots). On the other hand, *TaNAC2* was significantly upregulated at 2 hpi but downregulated at 8 and 20 hpi, after which it exhibited no changes in expression (Fig. 5H). Finally, the genes related to PR proteins (*TaBWPR1.2#2* and *TaPR10*) were significantly increased at 15 dpi in aerial biomass (Fig. 5H).

### Major RGE-based transcriptional-activity relationships.

**FIG 6:**
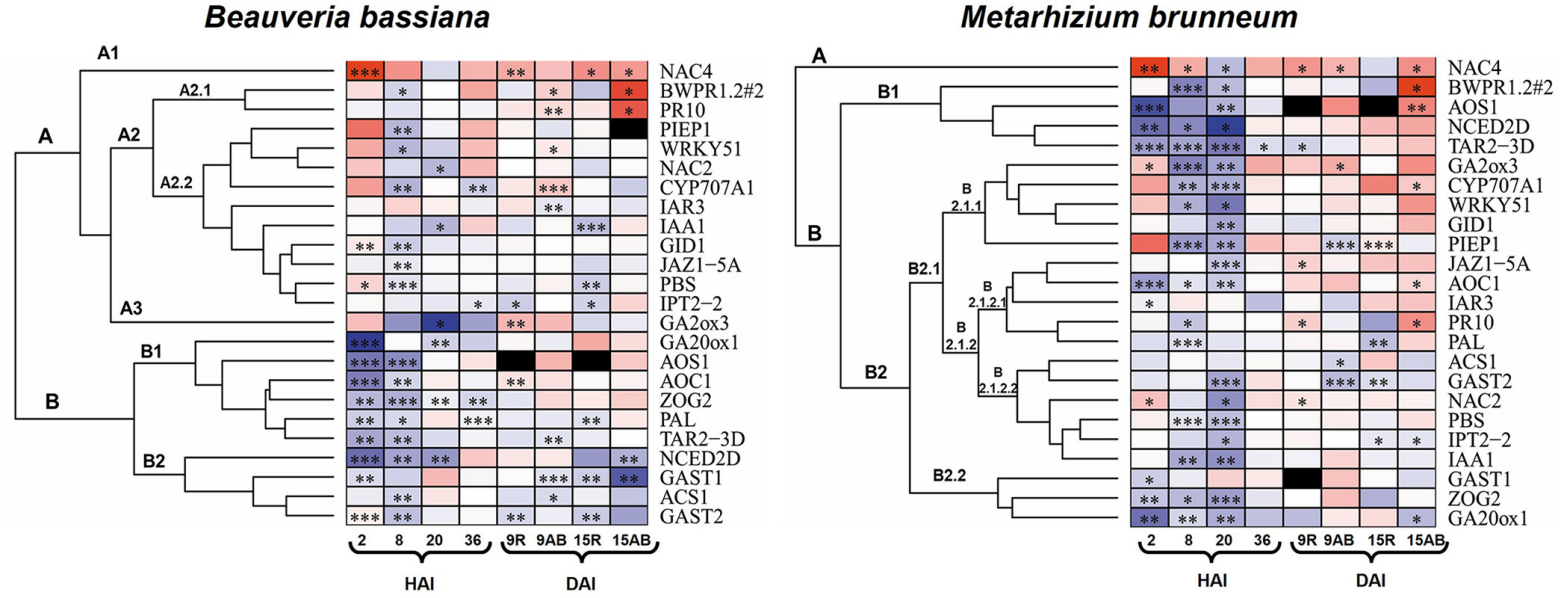
Heatmaps comparing relative gene expression (*n *= 3 biological replicates, 9 technical replicates) of the studied genes (names are given without “Ta”) in seedlings treated with B. bassiana and M. brunneum relative to their expression in control seedlings. Genes clustered in a dendrogram based on Pearson’s correlations. Data were obtained at three different times (2 to 36 h after inoculation [HAI] and 9 to 15 days after inoculation [DAI]) from roots (R) and aerial biomass (AB). The color scale goes from blue to red, meaning down- and upregulation, respectively. Darker hues represent higher RGE levels. *, *P < *0.05; **, *P < *0.01; ***, *P < *0.001.

The RGE values for all genes studied were used to construct a heatmap (Fig. 6) and a correlation matrix (Fig. S4) for each fungus in order to identify patterns or cross talk between hormones. The heatmaps revealed marked downregulation of most of the genes during the colonization time with both fungi (Fig. 6).

The transcription factors (TFs) for pathogen-related genes grouped together at the top and hormone biosynthesis genes grouped at the bottom in the B. bassiana heatmap. Thus, the B. bassiana heatmaps exhibited two clusters consisting of 14 and 10 genes (Fig. 6, left, groups A and B, respectively). There were no changes in the first group during the colonization time except for downregulation at 8 hpi (Fig. 6, left, A). In contrast, group B changed throughout colonization (Fig. 6, left). Subgroups A1 and A2.1 comprised *TaNAC4*, *TaBWPR1.2*, and *TaPR10*, which fell very close to one another mainly by reason of their being upregulated in aerial biomass. In addition, PR protein genes were highly positively correlated with one another and also with *TaIPT2-2* and *TaIAA1* (Fig. S4). *TaGAST1*, *TaGAST2*, and *TaACS1* also clustered: they were the most consistently downregulated genes (Fig. 6), as confirmed by the high positive correlation between *TaGAST1* and *TaACS1* (Fig. S4). Finally, genes *TaNAC4*, *TaWRKY51*, *CYP707A1*, and *TaPBS1* were highly positively correlated, which suggests cross talk between them.

Except for *TaPR10*, the transcription factors for pathogen-related genes also grouped together in the M. brunneum heatmap but seemingly correlated with jasmonate, auxin, and abscisic acid biosynthesis genes, as well as with those of abscisic acid and gibberellin catabolism. The heatmap also comprised two clusters: one (Fig. 6, right, group A) for *TaNAC4*, which was mainly upregulated throughout, and the other encompassing all other genes (Fig. 6, right, group B). Worth special note was subgroup B1 (*TaBWPR1.2#2*, *TaAOS1*, *TaNCED-2D*, and *TaTAR2-3D*), which exhibited initial downregulation during the colonization time but upregulation at 9 and 15 dpi. Subgroup B2.2 comprised *TaGAST1*, *TaZOG2*, and *TaGA20ox1*, which fell together in the heatmap because they were repressed during the colonization time (especially at 2 hpi). There was interesting cross talk between some genes, such as *TaWRKY51*, *TaPAL*, *TaBWPR1.2#2*, and *TaPR10*, which were significantly positively correlated with one another and with *TaTAR2-3D* (*TaPAL* excepted), *TaGID1*, and *TaGA2ox3* (Fig. S4). Finally, judging by their significant positive correlations, there was seemingly close cross talk between SA- and GA-related genes and between JA- and auxin-related genes (Fig. S4).

## DISCUSSION

### Effect of EF inoculation on wheat growth.

The response of wheat to EF inoculation in terms of physiological changes was similar to that found in previous studies (29). Thus, there was an initial slowdown in growth that was subsequently overcome. In this work, however, plant growth was slightly more constrained than it was in previous studies with the same crop and fungal strains (22, 30), as can be seen, for example, by the yield or root length results. The differences may have arisen from the procedure followed here, in which fungal inoculation was done by seedling immersion in a conidia suspension of 1 × 10^8^ conidia/mL and real soil was replaced by perlite. These two factors boosted fungus-plant interactions. Thus, treating seedlings instead of seeds increased the contact surfaces through immersion and also created conidium reservoirs in the plant tissues that could keep conidia ungerminated until several weeks after. Besides that, perlite does not retain conidia as much as real soil. Therefore, this causes higher availability of fungal conidia, which can constrain the plant after longer periods of time, as has been shown by the ADM, RDM, and height parameters at 67 dpi (with B. bassiana).

### Wheat immune system at EF colonization time.

Our results fitted the zig-zag model of activation of the plant immune system (31). The first stage involves the initial recognition of the fungi by microbe-associated molecular patterns (MAMPs) and pathogen-associated molecular patterns (PAMPs), which is linked to the RGE results obtained at 2 hpi. Pattern recognition receptors (PRRs) trigger PTI (pathogen-triggered immunity), which in turn triggers JA/ET-dependent induced systemic resistance (ISR) (32). This is consistent with the increased RGE in the transcription factors *TaNAC2* and *TaNAC4*—*TaNAC4* overexpression is induced by methyl jasmonate (MeJA), ABA, and ET but not by SA (33), which is consistent with the absence of systemic acquired resistance (SAR). *TaNAC2*, whose expression is induced by abiotic stresses (34), is also involved in the initial recognition of MAMP or PAMP; however, as shown here, *TaNAC4* is the actual TF induced by biotic stresses (33). Triggering of ISR rather than SAR is also supported by the fact that the PR gene *TaBWPR1.2#2* and the ethylene response factor (ERF) *TaPIEP1* were upregulated by JA and JA plus ET, respectively (35–37). Overexpression of *TaWRKY51*, which is a positive regulator of ISR in barley, is consistent with active cross talk between SA and JA (38). On the other hand, the significant increase in *TaGID1* (mainly with B. bassiana at 2 hpi) is consistent with stabilization of the DELLA protein complex, which downregulates the GA signaling pathway (39), consistent with upregulation of *TaGA2ox-D9* and downregulation of *TaGA20ox1*. Thus, the DELLA protein complex is not degraded and can hijack *TaJAZ1-5A* (40), a repressor of the JA signaling pathway. However, genes related to the synthesis of JA (*TaAOS1* and *TaAOC1*) and ET (*TaACS1*) were strongly downregulated, which suggests that triggering of hormonal defenses is suppressed in an unknown manner.

The transcription factor *TaGAST1* was slightly downregulated through suppressed synthesis of ABA and GA, which are two positive regulators (41). This is consistent with the increased expression of genes encoding catabolic enzymes of ABA (*TaCYP707A1*) and GA (*TaGA2ox-D9*) and with the decreased expression of those involved in their synthetic pathways (*TaNCED-2D* and *TaGA20ox1*, respectively). On the other hand, *TaPBS1* overexpression at 2 hpi, which was significant with B. bassiana, was a result of the kinase protein encoded by this gene being involved in PTI signaling (42).

The second stage of the immune system response starts with induction of effector-triggered susceptibility (ETS) by the effectors from successful microorganisms, which in turn suppresses pathogen-triggered immunity (PTI) (31, 32). In response, plants develop effector-triggered immunity (ETI) in the third stage. Based on the differential expression pattern observed at 2 hpi, the results of the gene analyses performed at 8 and 20 hpi can be assigned to the previous two stages. Beauveria bassiana and Metarhizium brunneum effectors may suppress the immune response (43) to facilitate colonization of plants, inducing systemic resistance (44–47). This hypothesis is supported by the fact that both EF downregulated PR genes (*TaBWPR1.2#2* and *TaPR10*) at 8 hpi and suppressed the SA-JA (*TaWRKY51*) and JA/ET (*TaPIEP1*) signaling pathways and highly sensitive transcription factors like *TaNAC4*. Subsequently (20 hpi), the plant immune system recovered slightly (*TaPAL*, *TaAOC1*, and *TaACS1*), thus bringing ETS and ETI triggering to an end (third stage). Surprisingly, *TaIAR3* was slightly upregulated at 8 and 20 hpi, possibly as a result of auxins being synthesized through an alternative or additional pathway under stress conditions (48).

The similarity of the RGE patterns observed at 36 and 2 hpi suggests that delivery of other microbial effectors might suppress SAR and trigger the JA signaling pathway. This assumption is consistent with the increased expression of *TaAOS1* and *TaJAZ1-5A* with B. bassiana and the triggering of ABA signaling (*TaCYP707A1* and *TaNCED-2D*), which contradicts the SAR response (49). However, this response was less marked than the previous one and suggests also less marked expression of the *TaPBS1* gene, so the kinase protein receptor may be more essential to PTI than it is to ETI (42). On the other hand, triggering of the ABA signaling pathway—mainly with B. bassiana at 36 hpi—may be a way of inducing stomatal closure (43) to prevent further fungal colonization of the tissue (50). This is consistent with the generally decreased colonization rate of B. bassiana relative to that of M. brunneum even though the EF’s effectiveness was similar to that found in previous studies (30).

The decreased growth observed here from 9 to 19 dpi was seemingly a consequence of the need for the plants to invest energy in defense mechanisms and in fighting initial stress (51). Thus, synthesizing secondary metabolites to supply the immune system had a cost and detracted from plant growth (51), an assumption consistent with the downregulation of *TaTAR2-3D* (involved in auxin synthesis) at early stages. This hypothesis is supported by the previous results of Zhang et al. (37), who found the cost associated with the synthesis of proteins from the PR family (*TaPR10* and *TaBWPR1.2#2*) to be closely related to the JA response (52), which supports the positive correlation with the presence of M. brunneum found here. The other hypothesis put forward to explain the initial growth constraint involves translocation of carbon from plants to fungi (32, 51, 53), with plants prioritizing photosynthetic organs to offset the sink, as suggested by the significantly increased ADM/RDM ratio with both fungi at 36 dpi, immediately after the differences in growth peaked.

Triggering of ISR (i.e., increased *TaAOS1* and *TaAOC1* expression levels), which occurred with both fungi at 15 dpi (mainly) here, was previously observed in maize inoculated with Metarhizium spp. at the same growth stage (2 weeks) (27). Therefore, plant-fungus cross talk under controlled conditions may have a similar duration and also follow similar patterns irrespective of the crop (possibly as in the zig-zag model). Besides the differences seen through time, wheat tissues exhibited differences in gene expression patterns. Thus, the translocation capabilities of EF (54, 55) may explain the increased activation of ISR in aerial biomass relative to that in roots, but one cannot discard the possibility of a delay in conidial germination in leaves as a result of seedlings being completely immersed in the fungal suspension.

Finally, ISR triggering was a growth-promoting effect, especially with M. brunneum (56). Plant growth is directly dependent on auxins, cytokinins (CKs), and GAs, which are responsible for cell growth, division, and elongation, respectively. Biosynthesis of CK and GA was inhibited until 15 dpi, when downregulation of *TaGA2ox-D9* and upregulation of *TaGA20ox1* by B. bassiana restored growth from 19 dpi. The delay can be ascribed to a timing difference between molecular and physiological responses. Even though GA and CK did not follow this trend with M. brunneum, the highest upregulation of *TaTAR2-3D*, at 15 dpi, signaled the time when the initial fungal constraint was overcome by successfully synthesizing auxin.

### Conclusions.

Beauveria bassiana and Metarhizium brunneum depress the immune system of bread wheat to colonize its seedlings. This triggers an immune response that fits well with the model of Jones and Dangl (31). Initially, the fungus-plant interaction triggers induced systemic resistance (ISR) through interaction with biotrophs or herbivores. The amount of energy invested by wheat to trigger ISR may boost the development of photosynthetic tissues, as suggested by the substantial ADM/RDM ratio observed immediately after the fungally imposed constraint on growth peaked. The slowdown in physiological growth caused by the entomopathogenic fungi until 19 dpi was quite consistent with repression of genes that regulate the biosynthesis of hormones like auxin, gibberellin, and cytokinin. On the other hand, growth recovery in fungally treated plants from 19 dpi can be explained at the molecular level at 15 dpi and ascribed to a transcriptional increase in the genes encoding auxin, GA, and CK.

## MATERIALS AND METHODS

### Plant and fungal material.

Seeds of Triticum aestivum L. cv. Átomo were disinfected with NaClO and ethanol and washed with sterile deionized water as described elsewhere (29) for pregermination on wet sterile filter paper for 3 days and inoculation with entomopathogenic fungus (EF) Beauveria bassiana or Metarhizium brunneum. The resulting seedlings were placed in a controlled growth chamber at 65% relative humidity with a 16-h/8-h light/dark photoperiod and a day and night temperature of 24 and 18°C, respectively. The EF strains used were B. bassiana (Balsamo) Vuill. EABb 04/01-Tip and M. brunneum (Petch) EAMa01/58-Su from the Entomopathogenic Fungus Collection of the Agricultural Entomology Unit of the Department of Agronomy of the University of Cordoba (Spain). The two strains are registered in the Spanish Collection of Culture Types (CECT) with identification numbers 20744 and 20784, respectively. Both were allowed to multiply, and their conidial concentrations adjusted according to González-Guzmán et al. (22).

### Experimental design.

Two different experiments were performed. One lasted until 98 days postinoculation (dpi) (specifically, until maturation, 92 on Zadok’s scale) and was used to estimate the time needed by the fungi to colonize wheat and cause the greatest changes in growth. In the other, plants were sampled at times chosen in terms of the results of the previous experiment to examine wheat hormone and immune system patterns associated with fungal colonization and initial growth differences between treatments.

Three different treatments were used, namely, (i) no fungus (control treatment), (ii) 1 × 10^8^ B. bassiana conidia mL^−1^ plus 0.5% Tween 80 (vol/vol), and (iii) 1 × 10^8^ M. brunneum conidia mL^−1^ plus 0.5% Tween 80 (vol/vol).

A total of 56 3-day-old seedlings were immersed in 50 mL of each treatment (control or B. bassiana or M. brunneum suspension) in the first experiment and 36 seedlings in the second, the suspensions being stirred on a rotational shaker at 2 Hz for 45 min. Then, the seeds were sown in cylindrical polyvinyl chloride (PVC) pots 15 cm tall and 5 cm in diameter that were filled with perlite previously autoclaved twice at 121°C with an interval of 24 h. The pots had a filter-plugged 5-mm hole in the bottom to facilitate drainage. Seedlings were watered on alternate days with sterile deionized water and Hoagland nutrient solution [5 mM (NO_3_)_2_·4H_2_O, 5 mM KNO_3_, 2 mM MgSO_4_, 0.1 μM KCl, 0.3 μM Ca(H_2_PO_4_)_2_·H_2_O, 50 μM H_3_BO_3_, 4 μM MnSO_4_·H_2_O, 4 μM ZnSO_4_·7H_2_O, 10 mM Fe as ethylenediamine-*N*,*N*′-bis(2-hydroxyphenylacetic acid) (EDDHA), 0.1 μM CuSO_4_·5H_2_O, and 6 μM Na_2_MoO_4_].

### Fungal colonization of plants.

Fungal colonization was assessed in both experiments, with three samplings (12, 30, and 48 h postinoculation [hpi]) in the first and two (12 and 36 hpi) in the second. Six seedlings per fungal treatment per sampling time were used in each experiment. Seedlings were externally disinfected, chopped, and placed in four petri dishes (six pieces per petri dish, one piece belonging to one plant) according to González-Guzmán et al. (22). A total of 54 seedlings were thus analyzed in the first experiment and 36 in the second.

### Monitoring plant growth: height, dry matter, and root architecture.

Plant height was measured with a ruler 7 times until data exhibited an asymptotic curve. In contrast, biomass was determined 8 times by cutting 4 plants and splitting the material between root (R) and aerial biomass (AB). Grain yield was assessed at 98 dpi in 6 plants per treatment.

At each sampling time, roots were carefully washed and root length (RL) and root surface area (RSA) determined by image scanning, using an Epson scanner at 300 ppi and the software WinRhizo. Then, root dry matter (RDM) and aerial dry matter (ADM; leaves and stems) were determined after drying out the plant material in an oven at 60°C until weight constancy (72 h). The target parameters included the ADM/RDM ratio, specific root length (SRL, RL × RDM^−1^), and specific root area (SRA [RSA × RDM^−1^]). A total of 114 plants [(8 samplings × 3 treatments × 4 replicates) + (1 sampling × 3 treatments × 6 replicates)] were used to assess plant growth.

### RNA extraction and cDNA synthesis.

The hormone and immune molecular patterns associated with fungal colonization were elucidated in three biological replicates collected at 2, 8, 20, or 36 hpi. Each of these replicates was run in three technical replicates. An identical number of replicates collected at 9 and 15 dpi (54 plants in all) were used to examine physiological changes by targeted transcriptional analysis. The seedlings taken at 9 and 15 dpi were split into root and aerial biomass to evaluate differences between the two types of tissue. Plants were collected from the pots and washed three times with sterilized-deionized water, blotted with sterilized filter papers, shock-frozen in liquid nitrogen, and stored at −80°C until RNA extraction. Table 2 lists the genes associated with wheat hormonal metabolism (biosynthesis, catabolism, and conjugation) or immune response (plant stress regulation). After vegetal frozen tissues were crushed in a ball mill, plant RNA was extracted by using TRIzol (Invitrogen; Carlsbad, CA, USA). Then, RNA was precipitated with chloroform and isopropanol (200 and 500 μL, respectively) and carefully washed with 75% ethyl alcohol (EtOH)/diethyl pyrocarbonate (DEPC) water. Finally, RNA was dissolved in DEPC water. The DNA contamination was removed with the DNase treatment from Quantabio (Beverly, MA, USA) for 30 min at 37°C before adding 200 μL of phenol-chloroform (1:1) and repeating the pelleting process of centrifugation and addition of ammonium acetate (5 M) and ethanol (100%) to finally resuspend the pellet in 20 μL of DEPC water. Finally, RNA was quantified with a NanoDrop 1000 spectrophotometer from Isogen (Hackensack, NJ, USA). Only high-quality RNA samples with *A*_260_/*A*_280_ ratios of 2.0 (±0.15) were used for RT-qPCR. cDNA was synthesized by treating 1 μg of total RNA per 20 μL of reaction mixture volume with the qScript cDNA supermix kit from Quantabio (Beverly, MA, USA).

**TABLE 2:** Name, related hormone, function, NCBI accession number, reference(s), and primers for each gene

Hormone	Gene name	Gene function	GenBank accession no.	Reference(s)	Primer	
					Forward (5′–3′)	Reverse (5′–3′)
Auxin	IAA1 (early-auxin responsive) (*TaIAA-1*)	Encoding auxin-responsive protein	AJ575098	57	GAGGGGCTATGAGGACACCATT	CTTCTCGGGGTCGGCGGCCGCGGCC
	Tryptophan aminotransferase (*TaTAR2-3D*)	Biosynthesis	KM078761.1	58	GCGAGTCTTCATCTTTGCGT	GCTGATCAGGAGAGACGACA
	Auxin amidohydrolase (*TaIAR3*)	Conjugation	AY701776.1	59	TGGTACAGTACAAGTGTCCAAACC	CATGAACTCCGCCTCCTTG

Gibberellins	Putative GA receptor (*TaGID1*)	Regulating stability of DELLA protein	EU417816	31	CGTTCGCCAAGAGCCTCATCA	AGCAGGTAGAAGCCGATGGTG
	Gibberellin-stimulated transcript (*TaGAST1*)	Gibberellin-induced transcript	GQ370009.1	32	ATGAAGTACTGCGGGCTGTG	CACTTGGGCCTCTTCCTGG
	Gibberellin-stimulated transcript (*TaGAST2*)	Gibberellin-induced protein	EU095332.1	32	GTTCCCTGTGGAGGTGATGG	TGTTGCAGTAGGTCAGGCAC
	Gibberellin 20-oxidase (*TaGA20ox1*)	Biosynthesis	FR716525	33	CGCTACTGCTCGGAGATGAG	AGTCGTTGCCCTCGAAGAAG
	Gibberellin 2-oxidase (*TaGA2ox-D9*)	Catabolism/inactivation	LN828687	34	AATGGGGCTTCTTCCAGGTG	CAGCGGTAGGAGTCGTTGAG

Abscisic acid	9-*cis*-epoxycarotenoid dioxygenase 2D (*TaNCED-2D*)	Biosynthesis	KX711892.1	35	GTGGTGCTCGACAAGGAGAA	CAGAGGTGGAAGCAGAAGCA
	ABA 8′-hydroxylase (*TaCYP707A1*)	Catabolism	AB714577, AB714579, AB714579	35	CAGGCCATCTTCTTCCAGCA	CTCCTGGGAAGACCTGCAAG
	Pathogenesis-related protein-1.2 (*TaBWPR1.2#2*)	Defense against pathogens	AB711115	36	TCGGCGAGAACATCTTCTGG	AGTTCTGCTTCTCGTCCACC
	Pathogenesis-related protein 10 (*TaPR10*)	Defense against pathogens	EU908212	37	GGTGGAGTCGACCTACAAGC	TGAAGATGGCCGTGACAGAC

Cytokinin	Adenosine phosphate-isopentenyltransferase (*TaIPT2-2*)	Biosynthesis	GQ202267	38	CAAAAGGGGGCGGAGGAAG	GTAGACCTGCATGGAGTCGG
	Cytokinin/zeatin *O*-glucosyltransferase (*TaZOG2*)	Conjugation	JN128597	38	TTCATGGGCTACCGCAACTT	CCCGTTCTTCACTTGCTCCT

Jasmonate	Allene oxide synthase (*TaAOS1*)	Biosynthesis	AY196004	39	CTTCTCCCGCCCATCTTTGT	GAACAGCAGGTTGTGGCATG
	Allene oxide cyclase (*TaAOC1*)	Biosynthesis	KF573524	40	TCGTCCCCTTCACCAACAAG	CTGTAGATGGCCTCGTAGCG
	Jasmonate ZIM-domain transcriptional repressor (*TaJAZ1-5A*)	Defense triggering	MH063272	41	TTCAGGGTTCAGAGTTCGCC	CAGCCACCGCATTGTTCATC

Ethylene	1-Aminocyclopropane-1-carboxylate synthase ACS1 (*TaACS1*)	Biosynthesis	U35779	42	GTCCCTGGATTTGGTGAGGG	GTATCCCGTCGTATGGCTGG
	WRKY51 transcription factor (*TaWRKY51*)	Lateral roots synthesis regulation	JX277054.1	44	GGATGGAGCACCTCATCCTG	AGTGATCTCCCGGCAGTCTA
	Pathogen-induced ethylene-responsive factor (ETR) (*TaPIEP1*)	Signaling	EF583940.1	45	GAACCACCACCTACCAGCAG	TCGAACGGAAGAACAGGTGG
	NAC transcription factor 2 (*TaNAC2*)	Defense signaling (abiotic stress)	AY625683	46, 47	GAGATGATGGCCACGCTGAT	GGATGTCGTCGTAGCTGAGG
	NAC transcription factor 4 (*TaNAC4*)	Defense signaling (abiotic stress)	HM027576.1	48	GTGACGGTGAAGGAGGACAG	TCACCATCTGCCCCATGTTC

Salicylic acid	Phenylalanine ammonia-lyase (*TaPAL*)	Biosynthesis	AY005474	43, 49	CGTCCTTGCTGAGGTCTTGT	GCTTCCCTCCAAGATGTGCT
	AVRPPHB susceptible1 (*TaPBS1*)	Signaling receptor	KY583249.1	50	AGGAACCGTTGGACTGGAAC	TGGTGGTTGTGCCTTGTCAT

### RT-qPCR analysis.

All 24 genes associated with regulation of plant growth or stress (Table 2) were analyzed by RT-qPCR. All primers were identified directly from the literature or designed by using Primer3Plus and ThermoFisher Primers Designer (Table 2). RT-qPCR was performed on a LightCycler 480 qPCR platform from Roche Diagnostics, Ottweiler, Germany, using reaction mixtures containing 2.5 μL of SYBR green master mix (2×), 0.25 μL of forward and reverse primers (0.25 μM), and 2 μL of cDNA. The mixtures were prepared in final volumes of 5 μL by using a Janus robot. The protocol comprised four steps, namely, preincubation, amplification (45 cycles), melting curve and cooling. All genes were normalized against the housekeeping genes (HKGs) actin gene and *Ta2291* (ADP-ribosylation factor) according to Paolacci et al. (57). The relative fold differences for each gene were assessed by normalizing the cycle threshold (*C_T_*) value for the gene against those for both HKGs, and relative gene expression (RGE) was calculated with reference to a calibrator (control) by using the 2^−ΔΔ^*^CT^* method (58).

### Statistical analysis.

Dry matter, height, root architecture, and RGE data were analyzed by comparing the means of the replicate values for the control seedlings and those inoculated with B. bassiana and M. brunneum separately through Student’s *t* test. Data not fulfilling the assumptions of a parametric test were subjected to the Kruskal-Wallis test. Herein, the term “significant” means a *P* value of <0.05. All statistical analyses were done with the software Statistix version 10 from Analytical Software (Tallahassee, FL, USA). In addition, two heatmaps (one per EF) were constructed from the RGE values to obtain an overall view of gene up- and downregulation in treated seedlings relative to untreated ones during the colonization time (first 36 hpi), as well as in roots (R) and in aerial biomass (AB) at 9 and 15 dpi. Pearson’s correlation coefficients between RGE values for treatment with each EF were determined to check for potential cross talk signaling between genes. The *heatmap.2* and *gplots* functions were used for heatmapping, as was *corrplot* for Pearson’s correlation analysis, all in the software RStudio version 1.2.5033 (59).

### Data availability.

Data are available by request. Primers and accession numbers of genes are in Table 2.
