# Supplementary material for: Elucidating the Effect of Endophytic Entomopathogenic Fungi on Bread Wheat Growth through Signaling of Immune Response-Related Hormones
Source: Appl Environ Microbiol. 2022 Aug 29;88(18):e00882-22. doi: 10.1128/aem.00882-22 (PMC9499012; doi:10.1128/aem.00882-22)
Supplement: Supplemental file 1 — Fig. S1 to S4. Download aem.00882-22-s0001.pdf, PDF file, 0.3 MB [file aem.00882-22-s0001.pdf]

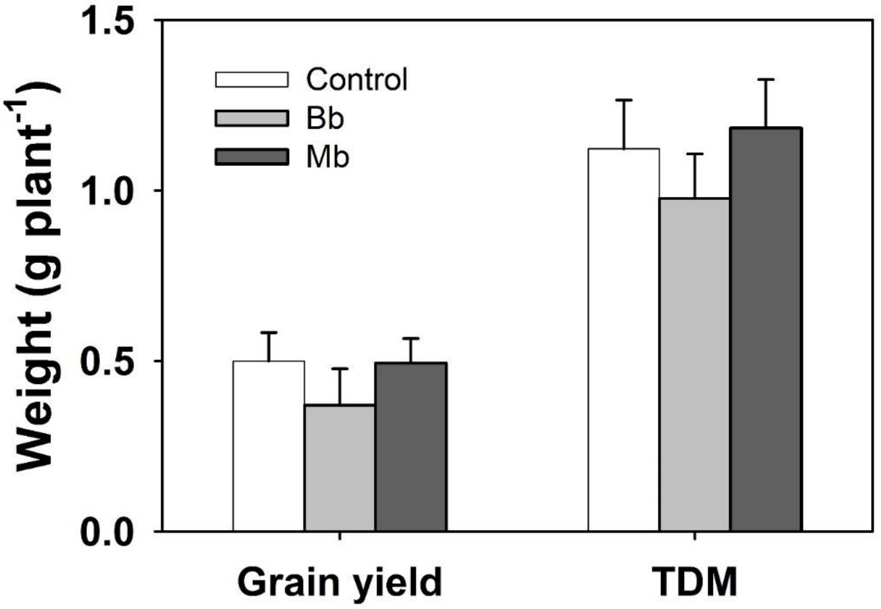

Figure S1: Grain yield and total dry matter (TDM) of the wheat plants treated with *B. bassiana* (Bb), *M. brunneum* and untreated ones (Control) (n = 6) at harvest (98 DAI) in the experiment 1. The lack of asterisks (*) indicates no significant differences.

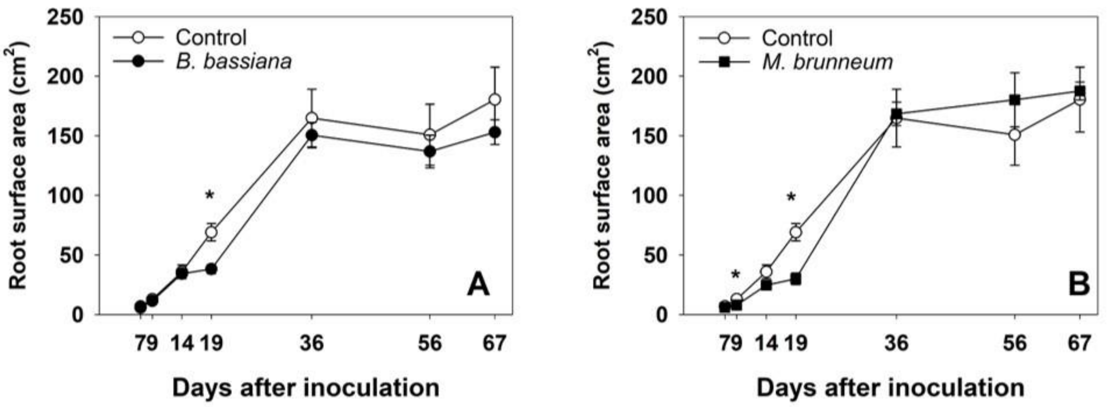

Figure S2: Root surface (RSA) monitoring throughout the crop cycle comparing control against *B. bassiana* (A) or *M. brunneum* (B) in experiment one. One asterisk denotes significant differences at $P < 0.05$.

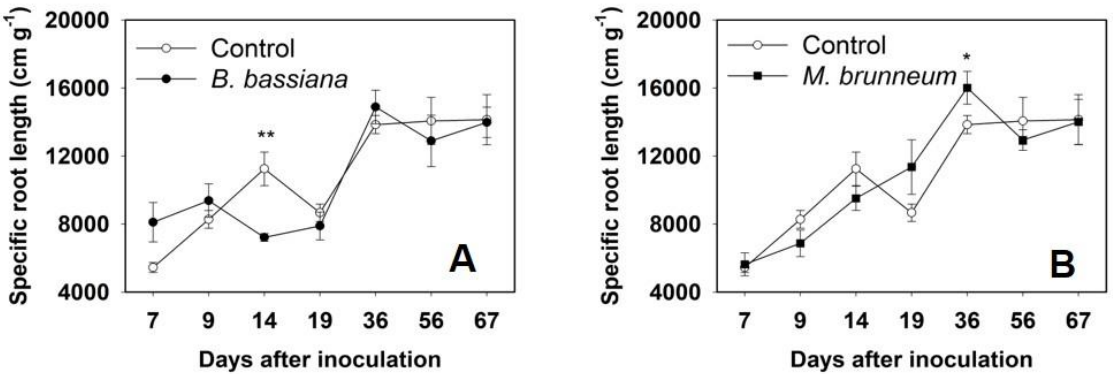

Figure S3: Specific root length (SRL) monitoring throughout the crop cycle comparing control against *B. bassiana* (A) or *M. brunneum* (B) in experiment one. One asterisk denotes significant differences at $P < 0.05$.

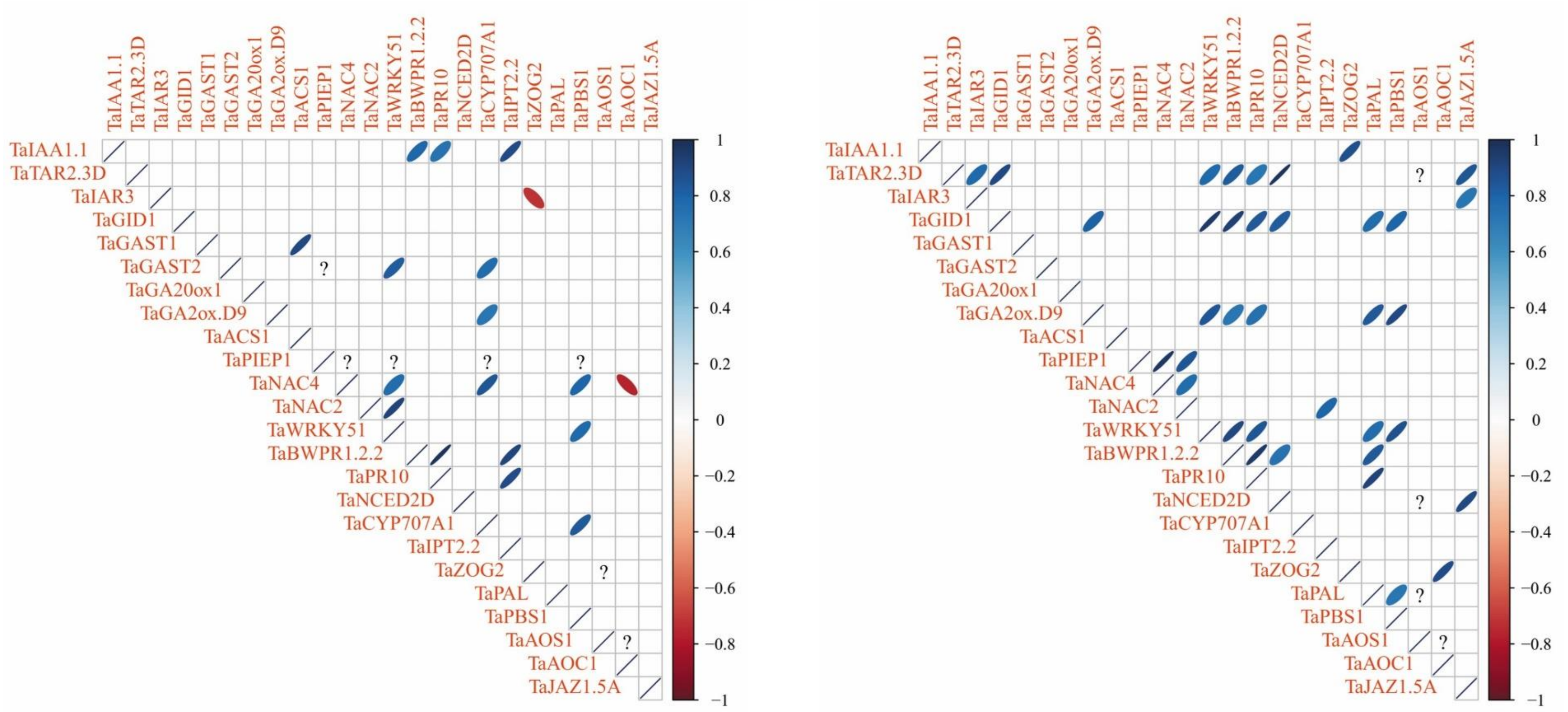

Figure S4: Pearson correlations between relative gene expression of plants treated with *B. bassiana* (left) or *M. brunneum* (right) (n = 8). Blue and red ellipses mean positive and negative correlations, respectively. As much as thin is the ellipse higher is the correlation between the genes. These two-correlation graphic only represent the significant correlations ($p < 0.05$).
